# How Do Cognitive and Motor Dual-Tasks during Small-Sided Games Impact the Tactical Performance of Youth Soccer Players?

**DOI:** 10.5114/jhk/192202

**Published:** 2024-12-19

**Authors:** Pedro Emílio Drumond Moreira, Maicon Rodrigues Albuquerque, Leonardo de Sousa Fortes, Gibson Moreira Praça

**Affiliations:** 1 Sports Department, Universidade Federal de Minas Gerais, Belo Horizonte, Brazil.; 2 Neurosciences of Physical Activity and Sports Research Group, Universidade Federal de Minas Gerais, Belo Horizonte, Brazil.; 3 Department of Physical Education, Federal University of Paraíba, João Pessoa, Brazil.

**Keywords:** cognitive performance, motor performance, metacognition, team sports, tactics

## Abstract

Numerous studies have investigated physical and motor performance during dual-task protocols in different sports. However, only few studies have investigated tactical performance in dual-task situations in real-world game situations, such as small-sided games (SSGs). Moreover, sport-specific literature is unclear about the effects of motor or cognitive secondary tasks and the type of the stimulus (memorization, math operations) on players’ tactical performance. This study considered the impact of dual tasks on soccer players’ tactical performance within SSGs and compared the effects of motor and cognitive secondary tasks on soccer players’ tactical performance. A total of 24 U-13 soccer players (12.34 ± 0.55 years) took part in the study, playing SSGs under four different conditions: a single task (ST) condition: players performed only SSGs; a motor dual-task (MDT) condition: players performed SSGs while balancing a basketball ball on a cone; a cognitive dual-task 1 (CDT1) condition: players performed SSGs while doing math operations; a cognitive dual-task 2 (CDT2) condition: players performed SSGs while doing a memorization task. Tactical performance was assessed using the FUT-SAT under all experimental conditions. Players showed higher tactical performance in SSGs with a ST than SSGs with secondary tasks (p < 0.001). When comparing secondary tasks in SSGs, players presented a better tactical performance in SSGs with cognitive secondary tasks than in SSGs with a motor secondary task (p = 0.02). In conclusion, dual tasks impair the tactical performance of soccer players, with the motor secondary task inducing greater impairment than secondary cognitive tasks.

## Introduction

Players perform simultaneous actions throughout the game in team sports, such as soccer (Furley and Memmert, 2012). For example, the soccer player in possession has to dribble while visually tracking teammates looking for passing opportunities. Previous studies have attempted to replicate this demand in the literature by adopting dual-task protocols (Gabbett et al., 2011). These protocols are usually represented by the simultaneous requirement of motor and cognitive tasks. For example, a previous study requested players to juggle while engaging in math operations (Laurin and Finez, 2020). A previous systematic review showed that dual-task protocols increased the cognitive demand of players, impacting cognitive and motor performance in lab contexts (Moreira et al., 2021). The study by Tapper et al. (2017) showed a decrease in performance of the multiple object tracking task (3D-motion) in dual-task situations (accuracy in the multiple object tracking task during a performance task with an acoustic requirement). However, in game-based tasks, these effects are unknown.

The engagement in two simultaneous tasks can be understood under the dual-process theory that assumes two sources of processing in the control of human behavior: automatic and controlled (Evans and Stanovich, 2013). The automatic system is fast and low-attention demanding, usually prevalent in automatized situations. On the other hand, the controlled system is characterized by being slower and high-attention demanding. For example, in a soccer match, the player decides to pass the ball to teammates in a good position, which is done with low attentional resources allocated in the motor execution (type 1 processing). Simultaneously, attentional resources are allocated to recollecting the strategic plans of overcoming the opponent who is in the position that the teammate is positioned (type 2 processing) (Furley et al., 2015). The addition of a secondary task activates the controlled system, increasing attentional demand (Furley et al., 2015). Based on the limited-capacity attentional resource (Abernethy et al., 2007), the overload generated during dual-tasks impairs cognitive processing related to memory and attention, which would reduce performance (usually known as “cost”) under the dual-task paradigm. However, there is no available information in the literature on whether adding a secondary task in game-based tasks (such as SSGs) impairs players’ tactical performance. This knowledge will be helpful to practitioners when designing training tasks and deciding whether or not to adopt dual-task training in their routines. According to the taxonomy of dual tasks: “dual tasking is the concurrent performance of two tasks that can be performed independently, measured separately, and have distinct goals” (McIsaac et al., 2015). Therefore, it could be assumed that playing an SSG represents a simple task (one goal), and including the secondary task (cognitive or motor) changes it into a dual-task situation.

Previous studies adopted mainly physical and motor outcomes to analyze the impact of dual tasks on performance (Gutiérrez-Davila et al., 2017; Laurin and Finez, 2020; Prończuk et al., 2023, 2024; Skalski et al., 2024). The study of Gutiérrez-Davila et al. (2017) investigated the reaction time of fencers’ attack under a dual-task condition and found a lower reaction time under the single-task condition than in the dual-task. However, performance in soccer is also influenced by the player’s ability to solve emerging problems, i.e., tactical action (Bar-Eli and Raab, 2006). At this point, as most studies focused on technical and physical dependent variables, little is known about the impact of dual tasks on decision-making skills (Moreira et al., 2021). Furthermore, the few studies (cited in the review study of Moreira et al., 2021) that analyzed decision-making skills were restricted to laboratory contexts, limiting the comprehension of dual-task impacts on sports contexts.

In soccer, the decision-making process is complex and involves integrating cognitive and motor tasks. Players need to quickly assess perceptual information from the environment, such as the positions of opponents and teammates, while simultaneously planning and executing appropriate motor actions, such as passes, dribbles or shots (Roca et al., 2012). This dynamic process requires selective attention to filter relevant information, as well as rapid analysis of constantly changing scenarios, enabling players to make quick and effective decisions during the game (Hüttermann and Memmert, 2017). To the best of our knowledge, no study has explored the influence of different types of secondary tasks (secondary cognitive and secondary motor tasks) on players’ decision-making in dual tasks. This knowledge will help practitioners better choose between secondary motor and cognitive tasks when adopting dual-task training in soccer. Based on the information processing model of attention (Wickens, 2021), the type of the secondary task can influence the cost during dual-task protocols. For example, a visual secondary task is supposed to impair driving performance more prominently than an acoustic secondary task, as driving relies more on visuospatial attention (Broeker et al., 2020). In sports, the players’ actions depend on decisional processes and motor actions (Weigel et al., 2015). Consequently, it might be expected that secondary motor and cognitive tasks will present similar impacts on in-game performance, contrary to laboratory studies in which the components (cognitive and motor) were investigated in isolation (Fleddermann et al., 2019; Fleddermann and Zentgraf, 2018). However, to the best of our knowledge, no study has tested whether motor or cognitive secondary tasks impact players’ performance differently.

Soccer performance analysis usually involves observing players’ space management and cooperation-opposition relationships in game-based tasks. For example, the System of Tactical Assessment in Soccer (FUT-SAT) (Teoldo et al., 2011) is a commonly adopted observational tool that covers core tactical principles of the soccer match and provides a performance score based on the percentage of correct tactical actions (decision-making assessed as appropriate within the match situation). Recent studies adopting the FUT-SAT analyzed tactical performance over time (Praça et al., 2017b), expert-based differences (Da Silva et al., 2021), as well as the influence of cognitive effort (Cardoso et al., 2019). Therefore, a potential sensibility of the instrument to detect dual-task cost in small-sided games (SSGs) can be assumed. SSGs represent a scenario facilitated and representative of the game of soccer for younger players (Bělka et al., 2023), allowing the assessment of tactical performance under different conditions (Klingner et al., 2022).

Coaches aim to reproduce the demands arising from the game in the training scenario, which is a dual-tasking condition. Understanding the impacts of dual tasks on different stimuli (motor and cognitive) on tactical performance can be important for adjustments in cognitive load tasks. Furthermore, the present study may contribute to understanding the chronic effects of dual-task training on the tactical variable. In this regard, the current study had two main aims. Firstly, we compared youth soccer players’ tactical performance (percentage of correct tactical actions) throughout SSGs played under single- and dual-task conditions (two secondary cognitive tasks and one secondary motor task). According to the limited-capacity attentional resource (Abernethy et al., 2007), we expected a lower performance under dual-tasks than single-task conditions due to the generated information overload that impairs cognitive processing related to memory and attention, which is relevant to tactical performance. Secondly, we compared the cost of tactical performance between dual-task protocols with motor and cognitive secondary tasks. Based on the information processing model of attention (Wickens, 2021), we hypothesized that both tasks would impair players’ performance similarly since motor and cognitive actions are relevant to tactical performance in the context of SSGs.

## Methods

### Participants

The sample size estimation was conducted before the beginning of the data collection using G*Power 3.17 software (Faul et al., 2007). The lowest η^2^p in comparing tactical performance between single tasks and dual tasks, i.e., 0.15, based on results obtained in previous research (Moreira et al., 2021) was converted into f effect size (0.47) and included in G*Power software. The study design was set as ANOVA, repeated measures, within factors, adopting one group and four measurements. The alpha value was set at 0.05, and the power value was set at 0.80. It resulted in the recommended sample of at least ten soccer players.

Twenty-four U-13 soccer players (age: 12.34 ± 0.55 years; training experience: 4.21 ± 0.20 years) were recruited from two clubs (12 players each) to avoid the influence of the specific characteristics of a single club on the outcome of this study. Players who trained for at least six months at the club were selected. Athletes with injuries were excluded. All the players regularly engaged in national and state tournaments and had a weekly routine of three 2-h training sessions and one official match on average (classified as Tier 3: highly trained/national level (McKay et al., 2022)). The Ethics Committee of the Universidade Federal de Minas Gerais approved this study (approval code: CAAE 52770421.4.0000.5149; approval date: 04 March 2022), and we followed all the Helsinki Declaration guidelines (each parent or legal guardian signed written informed consent).

### Design and Procedures

A randomized and crossover design (two within factors, four experimental conditions) was used for the experimental component of the present study. The order of the experimental conditions was randomly allocated based on balanced permutations generated by a web-based computer program (www.randomization.com). This procedure reduces the probability of the effect of one condition on the other. The experimental conditions were applied two days in a row at each club. All experimental conditions were repeated twice each day to the different clubs, totaling eight repetitions by condition. The reliability of the repeated measures between each condition was analyzed by calculating the Intraclass Correlation Coefficient (ICC) (absolute reliability; two-way random (Koo and Li, 2016)). Results showed moderate correlation (ICC = 0.54; IC 95% = (0.30–0.72), F (47, 141) = 2.21, *p* < 0.001). SSGs and tasks during all visits were completed at 2 pm.

Participants were requested to refrain from strenuous exercise and caffeine consumption 24 h before testing. All trials were conducted on an artificial grass field at temperatures between 25°C and 28°C, with relative humidity between 28% and 87%.

### Experimental Conditions: Small-Sided Games

A three-a-side small-sided game was adopted as the standard format in the current study. All the games were played on a 36 x 27-m pitch (162 m^2^ per player) with natural grass for three minutes over two bouts on each protocol and were recorded using a JVC HD Everio GZ-HD520 digital camcorder for further analysis. All official rules of the modality were applied. Considering the reported influence of the level of the opposition (Folgado et al., 2014) and the playing position (Praça et al., 2017a) on players’ tactical behaviour, teams were composed of a defender, a midfielder, and a forward, balanced according to the technical staff evaluation, and kept constant over the whole data collection period. Four teams within each club, i.e., A, B, C, and D, were formed and engaged in all experimental conditions in randomized and balanced order. Teams A and B, composed of the players best ranked by the technical staff, played against each other over the whole data collection period (and the same occurred with teams C and D, composed of the lowest-ranked players). There were four experimental conditions, explained in detail below.

*A single task (ST) condition:* teams played the standard 3-a-side SSG. The goal for both teams was to score as many goals as possible. All the official rules of soccer were applied, including the offside.

*A motor dual-task (MDT) condition:* besides playing the standard 3-a-side game, players were required to balance a basketball in a cone with one hand during the whole SSG. If the player lost control of the basketball, he would be considered offside, and no game-related action would be allowed until he repositioned the basketball in the cone.

*A cognitive dual-task 1 (CDT1) condition*: besides playing the standard 3-a-side SSG, players were required to perform math operations (addition and subtraction). Immediately before the SSG, each player received a number (not equal to those received by the other players) between 60 and 99 from the main researcher. Throughout SSG, every twenty-three seconds, four researchers, positioned on the sidelines, raised during a 10-s interval a blue or a red pinny indicating to players the operation they should perform. The blue pinny represented a minus two (−2) operation and the red pinny indicated an addition (+3) operation. The operations were summed up over the whole SSG. Immediately after the end of the SSG, each player wrote the final number, after all the operations, on an individual clipboard positioned on the sidelines (previously assigned to each player). Players were instructed to write the answer as fast as possible and not to talk to each other after the SSG. For the AxB matches, the order of the pinnies’ colors was blue, red, red, blue, blue, red, and red. For the CxD matches, the order was: blue, red, blue, blue, red, red, and blue. The number and the order of the operations were kept constant over the bouts, while the initial number was different for each player on each trial.

*A cognitive dual-task 2 (CDT2) condition:* besides playing the standard 3-a-side SSG, players were required to memorize a sequence of seven letters (only consonants) during the game. All the procedures were like in the CDT1 protocol, except that players were required to memorize a sequence of letters instead of making math operations. Four researchers positioned at the sidelines raised a board containing a letter that players should memorize every twenty three seconds; the board was raised for ten seconds. Immediately after the SSG, players wrote the sequence of the letters on a clipboard available on the sidelines. The sequences of letters in the AxB and CxD matches in the first bout were L J W P N H F and R Q K J M K H, respectively, while in the second bout, the sequences were: F Z V L P H K and P N L H R M L, respectively.

**Figure 1:**
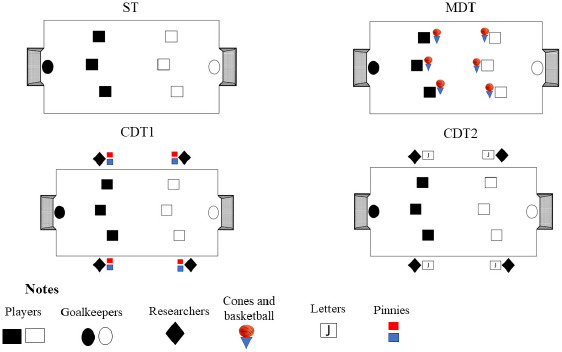
Experimental conditions. ST: a single task condition; MDT: a motor dual-task condition; CDT1: a cognitive dual-task 1 condition; a CDT2: a cognitive dual-task 2 condition

Figure 1 illustrates the experimental conditions. The study was conducted over one week in each club, without intervals between weeks. On the first day, players were familiarized with the protocols, explained all the doubts, and practiced the dual-tasks. Players engaged in the experimental conditions for two days, comprising eight bouts per team. The order of presentation of the protocols was randomized and balanced.

Each data collection started with a 10–min warm-up comprised of physical (jogging and sprinting) and technical (passing) activities. In the sequence, the three-minute bouts started for the AxB match and, in the sequence, for the CxD match. The data collection took approximately forty minutes daily (12 minutes of effective playing time for each team). There were three minutes of passive rest between the bouts for each team, assumed to be enough for the players to be recovered for the next bout (de Dios-Álvarez et al., 2023). One researcher was designed as a referee, responsible for controlling the game’s rules, the duration of the bouts, and the initial visual stimuli (math operations or memorization) when applicable.

### Instruments

The FUT-SAT was used to evaluate players’ tactical performance throughout SSGs (Teoldo et al., 2011). The assessment considered ten tactical principles (Offensive: penetration, offensive coverage, width and length, depth mobility, and offensive unity; Defensive: delay, defensive coverage, recovery balance, concentration, and defensive unity), which were judged by experts as successful or unsuccessful, following the criterion validated by the instrument. In addition, the percentage of positive tactical actions was adopted as the measure of tactical performance, as previously suggested in the literature (Moreira et al., 2020; Praça et al., 2017b).

The video files were transferred to a notebook (DELL®, Inspiron 14, series 3000). In the sequence, Soccer View 1.0 software (Teoldo et al., 2018) was used to insert the spatial references in the field and evaluate the tactical principles. After the analysis, 10% of the SSGs (Tabachnick and Fidell, 2007) were re-evaluated after twenty-one days from the first observation to ensure agreement within and between observers. Cohen’s Kappa coefficients were calculated for within-observer agreement, and Fleiss’ Kappa coefficients were calculated for the between-observer agreement (Robinson et al., 2007). The analysis showed acceptable between-observer (k = 0.77, *p* < 0.05) and perfect within-observer (k = 0.92, *p* < 0.05) agreements, as recommended by the scientific literature (Landis and Koch, 1977). R Studio software was used for these analyses.

### Statistical Analysis

Initially, the data were analyzed through descriptive statistics (mean and standard deviations). The assumptions of normality (Shapiro-Wilk’s) and sphericity (Mauchly’s) were tested in the sequence. A repeated-measures one-way analysis of variance (significance level set at *p* = 0.05) was used to compare the tactical performance among the four experimental conditions (ST x DT: first objective and secondary cognitive tasks x secondary motor task). A repeated-measures one-way analysis of variance was also employed for the second objective (comparing the cost of tactical performance among the three dual-task protocols). The following equation was used to measure the cost: tactical performance in the single task minus tactical performance in dual tasks divided by tactical performance in the single task, multiplied by 100 (Beurskens and Bock, 2012). The Tukey’s post hoc test was used for pairwise comparisons. Finally, the eta partial squared (η^2^p) was calculated to measure the effect size. The effect size was classified into no effect (η^2^p < 0.04), minimum effect (0.04 ≤ η^2^p < 0.25), moderate effect (0.25 ≤ η^2^p < 0.64) or strong effect (η^2^p ≥ 0.64) (Ferguson, 2009). The effect sizes of the pairwise comparisons were calculated through Cohen’s *d* and classified as small (0.2 ≤ *d* < 0.5), medium (0.5 ≤ *d* < 0.8), and large (*d* ≥ 0.8) (Cohen, 1988). SPSS 19.0 software (Statistical Package for Social Science) was used for all these analyses, except for Cohen’s *d* value, calculated with R Studio software.

Previous studies adopting the dual-task paradigm usually did not evaluate the performance in the secondary task (Howell et al., 2017; Laurin and Finez, 2020). However, this methodological issue might impair the interpretation of the data as it could not be assumed that individuals engaged in the second task. For example, they could maintain good performance in the main task by neglecting the execution of the secondary task during the execution, which would not allow for the interpretation of the dual-task effect. In the present study, the performance in the secondary tasks was measured through the magnitude of errors for each participant. In the CDT1 protocol, the difference between the final score reported by the participant and the expected value was assumed as the measure of the performance (the higher the difference, the poorer the performance). In the CDT2 protocol, the difference between the order of the letters and the expected sequence was assumed as the performance measurement. For example, if the expected sequence was L J W P N H F, but the participant reported the sequence L J W H R N F, the score of three (the three initial letters) was given to the participant. The final score was converted into a percentage to facilitate the interpretation.

## Results

**Figure 2:**
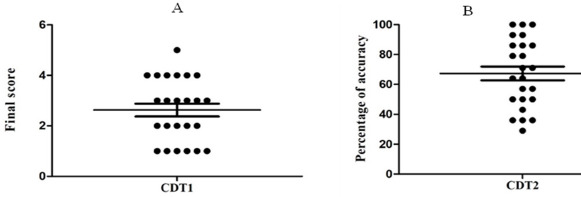
A) Scores on the secondary cognitive task in the CDT1 protocol. B) Percentage of accuracy on the secondary cognitive task in the CDT2 protocol.

Figure 2 presents the players’ scores on secondary cognitive tasks in the CDT1 and CDT2 protocols. The results showed values close to zero in the TDC1 protocol (Figure 2A), an accuracy rate of above 20% in the TDC2 protocol for all participants (Figure 2B), and an average accuracy rate of above 60% in this protocol, indicating that players engaged in the secondary cognitive tasks.

**Figure 3:**
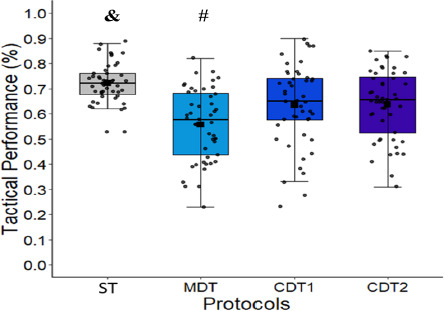
Comparative analysis of the tactical performance in the four protocols. ST: a single task condition; CDT1: a cognitive dual task (condition 1); CDT2: a cognitive dual-task (condition 2); MDT: a motor dual-task condition; &: significant difference (p < 0.05): ST > MDT, CDT1 and CDT2. #: significant difference (p < 0.05): CDT1 and CDT2 > MDT

Figure 3 shows the comparative analysis of the protocols. A main effect of the protocol was reported in the ANOVA (F (3,188) = 12.16, *p* = 0.001, η^2^p = 0.16). Participants showed higher tactical performance in the ST protocol (0.72% ± 0.80%) than in the MDT (55.73% ± 0.14%, *d* = 1.42), CDT1 (63.52% ± 0.16%, *d* = 0.68), and CDT2 (63.69% ± 0.13%, *d* = 0.75) protocols. Besides, participants also showed higher tactical performance in the CDT1 and CDT2 protocols than in the MDT protocol.

**Figure 4:**
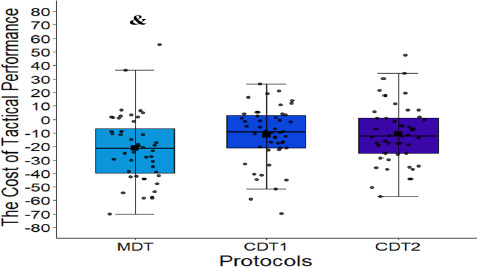
Dual-task cost in tactical performance. CDT1: a cognitive dual task (condition 1); CDT2: a cognitive dual task (condition 2); MDT: a motor dual task condition; &: significant difference (p < 0.05): MDT cost > CDT2 cost = worst tactical performance in MDT

Figure 4 shows the comparative analysis of the cost of tactical performance between dual-task protocols. A main effect of the protocol was reported in the ANOVA (F (2,141) = 3.10, *p* = 0.04, η^2^p = 0.04). Participants showed a higher cost of tactical performance in the MDT protocol (−21.10 ± 24.73) than in the CDT2 protocol (−10.64 ± 22.39, *d* = 0.44).

## Discussion

The current study aimed to compare the tactical performance of youth soccer players throughout SSGs played under single- and dual-task conditions and the cost of tactical performance between dual-task protocols with motor and cognitive secondary tasks. Performing dual tasks is typical in soccer and performance under these situations might be relevant for performance in the match (Gabbett et al., 2011), which justifies the current study. However, the sport-specific literature has not addressed the acute impact of dual tasks on tactical performance in soccer players. The current results indicate that players showed higher tactical performance under single- task than dual-task conditions (first aim). This confirms the initial hypothesis. Furthermore, for the second objective, the secondary motor task demonstrated a higher dual-task cost on tactical performance than the secondary cognitive tasks, contradicting the initial hypothesis.

Based on the limited information-processing resources (Abernethy et al., 2007), a high exigence in the secondary task induces a high attentional expenditure through the controlled system, which influences the control and maintenance of the automatic system, which is more related to the primary task (Gabbett et al., 2011; Laurin and Finez, 2020). At this point, assuming attention as an underlying process in the decision-making skill (Hüttermann and Memmert, 2017), it might be argued that the attentional requirements of the secondary tasks explain the biased tactical performance. It is suggested that this decrease in performance is mostly related to the difficulty in perceiving and interpreting relevant cues during the action—through the attentional process—which impairs the anticipation-decision coupling (Runswick et al., 2018). This argument is also supported by previous studies that observed delayed information processing time (higher reaction time) under dual-task conditions compared to single ones (Cochrane et al., 2019; Helm et al., 2016).

When comparing the MDT with the CTD2 protocol, a higher cost was observed in the motor secondary task protocol. The specificity of the secondary task proposed might explain this result. Specifically, previous studies suggest that secondary tasks not related to the primary ones cause distraction, increasing the cost of the dual tasks (Herold et al., 2018). In the MDT protocol, players were required to maintain (regulate) the balance of a ball on the mini cone through the body’s sensory pathway (e.g., motion adjustment when the individual pronates, high possibility of ball imbalance).

In contrast, the requirement in CDT1 and CDT2 protocols was that players solely needed to view out-of-field stimuli to complete the secondary task. Furthermore, it might be assumed that observing visual information around the pitch (e.g., CDT1 and CDT2 protocols) was closer to the cognitive demands of the soccer game (observing the coach gesturing some instructions and storing this information during the game) than balancing another ball in a cone. This, hence, might explain the current result. Finally, there is a clear gap in sport-specific literature when proposing both motor and cognitive secondary tasks with high specificity, which could lead to a higher transference to the actual context of the sport. Future studies should pursue this goal to analyze if secondary tasks with more specificity of sport induce higher costs in tactical performance.

The sample of the current study comprised U-13 soccer players. Previous studies showed that expert-novice differences were likely in dual-task protocols (Gabbett et al., 2011). Specifically, a higher level of cognitive skills (for example, improved working memory) might reduce the cost of the dual tasks in expert participants (Furley and Memmert, 2010; Furley and Memmert, 2012). However, at this point, it seems unlikely that the current sample has achieved an expert level. Therefore, the dual-task cost is still evident. This result reinforces the importance of investigating the current sample and understanding the role of dual-task training in decision-making development in youth sports.

The current study has several limitations. It was impossible to analyze players’ motivation and anxiety before and after the protocols, although these variables were reported to influence cognitive performance (Eysenck et al., 2007; Lang, 2016). Besides, the participants’ working memory was not measured, which reduces the possibility of understanding the mechanisms inherent to the dual-task cost in the present study. Thus, future investigations should aim to broaden the current findings by providing such information.

Considering the current results, suggestions for practical implications are possible. The use of dual-tasks in training can represent the demands of soccer in athletes’ decision-making processes. The need to shift attention to multiple points is relevant for perceiving crucial signals in decision-making. In this regard, dual tasks in SSGs can stimulate athletes towards external focus behaviors, also contributing to the movement automatization process. However, understanding the impacts of different types of dual tasks is fundamental for controlling the imposed cognitive load to avoid mental fatigue (Gantois et al., 2020). At this point, the initial selection of cognitive secondary tasks and later motor tasks can lead to load progression within the training process. Notwithstanding, these acute drops in performance are likely to be associated with long-term improvements in sports (Moreira et al., 2021). Therefore, the acute deleterious effect of including secondary tasks during SSGs might explain the long-term development of cognitive skills, which is usually the coaches’ target. At this point, previous studies on the subject demonstrate that exposure to dual-task training enhances cognitive processes underlying decision-making (Moreira et al., 2021; Wu et al., 2024).

## Conclusions

To the best of our knowledge, this is the first study investigating the impact of including secondary tasks during small-sided games on players’ tactical performance. It is concluded that the dual-task protocols lead to decreased tactical performance. Besides, it was shown that motor secondary tasks led to higher costs in tactical performance than cognitive ones.
